# Measuring Adult Health and Well-Being Outcomes Associated With Nature Contact in Parks and Other Forms of Protected Areas: Protocol for a Scoping Review

**DOI:** 10.2196/63338

**Published:** 2025-03-24

**Authors:** Jill Bueddefeld, Catherine E Reining, Loraine Lavallee, Ryan Brady, Mark W Groulx, Christopher James Lemieux

**Affiliations:** 1 Geography and Environmental Studies Wilfrid Laurier University Waterloo, ON Canada; 2 Department of Psychology University of Northern British Columbia Prince George, ON Canada; 3 School of Planning and Sustainability University of Northern British Columbia Prince George Canada

**Keywords:** nature contact, human health, mental health, well-being, parks, protected areas, outcomes

## Abstract

**Background:**

Growing evidence shows various health and well-being benefits from nature contact in parks and other forms of protected areas. However, the methods to measure these outcomes lack systematic identification, critical appraisal, and synthesis. Researchers working in this area would benefit from a clear framework highlighting key considerations when selecting measurement tools, along with a summary of the measures used, and insights into the limitations of generalizing existing research findings.

**Objective:**

The objectives of this scoping review are 2-fold. First, we aim to identify the instruments used to measure mental health and well-being outcomes of adults associated with direct nature contact in parks and other forms of protected areas. Second, we aim to evaluate the psychometric properties associated with the validity of these instruments to better understand the strengths and weaknesses of current measurement approaches.

**Methods:**

Following PRISMA-ScR (Preferred Reporting Items for Systematic Reviews and Meta-Analyses extension for Scoping Reviews) guidelines, 8 scholarly databases were searched (PubMed, Web of Science, PsycINFO [via ProQuest], ERIC [via EBSCOhost], CINAHL [via EBSCOhost], GreenFILE [via EBSCOhost], OVID, and GEOBASE) on January 4, 2023, for literature measuring the mental health and well-being outcomes associated with nature contact in protected areas. Sources were screened by reviewers based on clear inclusion or exclusion criteria relevant to the research questions: peer-reviewed English language studies measuring mental health and well-being focused on adults (aged 18+ years) with direct, in-person nature contact in parks and protected areas. Data will be extracted, analyzed, and represented according to 3 domains. This includes study details, characteristics of the measurement instruments, and their validity.

**Results:**

The results of the study and submission of a manuscript for peer review are expected in April 2025. The results of the scoping review are expected to contribute to an understanding of the diverse methods used to measure mental health and well-being related to nature contact in protected areas. Expected findings will include an organized summary of existing quantitative and qualitative instruments for measuring mental health and well-being outcomes, including appraisal of the instrument’s psychometric properties.

**Conclusions:**

To the authors’ knowledge, this will be the first scoping review undertaken on measures used to assess mental health and well-being outcomes related to nature contact in parks and protected areas context, offering a starting point from which to critically examine the validity and consistency of such methods. Findings will aid in identifying the strengths and weaknesses of current measurement approaches to mental health and well-being outcomes of nature contact and may be used to guide future research on this topic, helping researchers choose the best tool to assess outcomes.

**International Registered Report Identifier (IRRID):**

DERR1-10.2196/63338

## Introduction

### Overview

There is a growing body of research investigating the role of nature in human health and well-being, with numerous studies reporting improvements to physical, psychological, emotional, cognitive, social, and spiritual well-being from time spent in nature [1-4]. Within this context, parks and other forms of protected areas offer unique opportunities to connect with nature, and considerable research has highlighted the increasingly recognized health benefits afforded by these settings [5-8].

Globally, there are more than 295,000 protected areas covering 16.1% of the earth’s terrestrial or freshwater area and 8.2% of its marine area [9]. There is also a highly ambitious global initiative to protect 30% of earth’s land or freshwater and marine area by 2030, as per the United Nations Convention on Biological Diversity Kunming-Montreal Global Biodiversity Framework [10]*.* If this goal is achieved, the designation of parks and other forms of protected areas would represent the fastest and largest land or freshwater and ocean allocation in the history of the modern conservation movement. Protected areas differ from most urban green space and local-regional parks in that they have legislated management objectives to conserve nature and provide opportunities for human enjoyment. The International Union for Conservation of Nature (IUCN) defines a protected area as “a clearly defined geographical space, recognized, dedicated and managed, through legal or other effective means, to achieve the long-term conservation of nature with associated ecosystem services and cultural values” [11].

Protected areas can include national and subnational protected area designations, such as national parks, state or provincial parks, and a variety of other designations that fit within the IUCN definition. It is estimated that such areas receive more than 8 billion visits annually [12], underscoring their significance as an essential ecosystem service. It is also estimated that parks and protected areas provide health services valued at US $6 trillion annually worldwide (representing 8% of global gross national product) [13]. Evidence establishing the significance of protected areas to human health and well-being continues to grow at the same time that many nations are committing to significantly expand protected areas networks by 2030. Given these joint trends, this proposed review offers a timely stock take that will help direct the current state of knowledge toward a stronger evidence-informed practice related to the use of parks and protected areas as a nature-based mental health service.

In addition to being timely, the proposed review will provide an important new tool for researchers who face numerous challenges investigating the impact of nature on human health and well-being. Previous research has examined the impact of nature on human health, demonstrating that mental health and well-being outcomes are the most frequently studied [2]. Despite a growing wealth of research, steps to critically evaluate and provide decision support to researchers and practitioners at the level of instrumentation are lacking. The dearth of current decision support presents a critical gap as well-being is a wide-ranging concept, with still wider-ranging options related to measurement. Well-being can capture relatively transitory emotion and mood states, more stable aspects of positive identity such as self-acceptance and self-esteem, broad global evaluations such as subjective life satisfaction, and improvement in clinical conditions such as anxiety and depression.

The need for this review is underscored by a past systematic review conducted by Cooke et al [14]. While not specific to nature-based interventions, Cooke et al [14] identified more than 40 different instruments for measuring well-being that varied widely in length, psychometric properties (ie, validity), and use cases. Linton et al [15] argue that such variability may in part be due to a lack of agreed upon criteria of what an instrument should contain. As noted, despite the current variety, ongoing creation of new instruments, and proliferation of their use in nature-based contexts, there has been no systematic or critical examination of the methods used to measure health and well-being outcomes associated with nature contact in parks and other forms of protected areas. This is a critical knowledge gap that the proposed scoping review will address.

Researchers working in this area would benefit from a clear framework identifying the features to consider in measurement selection as well as a summary of the measures that have been used and their validity. A framework of this type would provide a clearer picture of aspects of well-being that have and have not been investigated or replicated and limits to the generalizability of the research to date. To support further research and decision-making related to outcome-based management in parks and other forms of protected areas, the proposed scoping review seeks to address two objectives: (1) to identify the instruments used to measure mental health and well-being outcomes of adults associated with direct nature contact in parks and other forms of protected areas and (2) to evaluate the psychometric properties associated with validity of the instruments used to measure mental health and well-being outcomes associated with direct nature contact.

### Existing Reviews

The current protocol was informed by an initial review of existing peer-reviewed literature to identify potentially comparable knowledge syntheses. To capture the current state of research related to mental health and well-being outcomes from nature contact, we gathered and documented scoping reviews published within the past decade that focused on an adult population. A search was conducted in Google Scholar to locate relevant scoping reviews, using variations of keywords that included “nature contact,” “mental health,” “well-being,” and “scoping reviews.” Table 1 outlines details of 7 relevant knowledge syntheses. All identified studies focused on constructs related to mental health and well-being. While 6 of the identified studies were specific to health and well-being outcomes related to nature contact, by contrast, none were specific to the unique context of parks and protected areas.

**Table 1:** Summary of comparable existing knowledge syntheses.

Citation	Title	Objective	Review of measurement instruments	Specific to outcomes from nature contact	Specific to parks and protected areas
Cooke et al (2016) [14]	Measuring Well-Being: A Review of Instruments	Identify and critically evaluate the psychometric properties of instruments measuring well-being and related constructs	Y^a^	N^b^	N
Wendelboe-Nelson et al (2019) [16]	A Scoping Review Mapping Research on Green Space and Associated Mental Health Benefits	Identify the variations across existing literature in the associations between green space and health benefits	Y	Y	N
Christiana et al (2021) [17]	A Scoping Review of the Health Benefits of Nature-Based Physical Activity	Summarize existing literature on the positive association between nature exposure, physical activity, and health outcomes	N	Y	N
Wilkie and Davidson (2021) [18]	Prevalence and Effectiveness of Nature-Based Interventions to Impact Adult Health-Related Behaviors and Outcomes: A Scoping Review	Document the use of nature-based interventions as a strategy to change adult health-related behaviors and outcomes	N	Y	N
Charles-Rodriguez et al (2022) [19]	The Relationship Between Nature and Immigrant’s Integration, Wellbeing and Physical Activity: A Scoping Review	Summarize existing research related to nature exposure, immigrant well-being, and physical activity	N	Y	N
Nejade et al (2022) [2]	What is the Impact of Nature on Human Health? A Scoping Review of the Literature	Summarize evidence relating nature-based interventions to health outcomes and examine enablers of nature contact	N	Y	N
Overbury et al (2023) [20]	Swimming in Nature: A Scoping Review of the Mental Health and wellbeing Benefits of Open Water Swimming	Summarize existing evidence relating to mental health and well-being benefits of open water swimming	N	Y	N

^a^Y: yes.

^b^N: no.

Cooke et al [14] reviewed 42 different instruments used to measure aspects of psychological well-being, psychosocial well-being, and psycho-physical well-being. The study categorizes these instruments according to 4 well-being categories (hedonic, eudaimonic, quality of life, and wellness) and a fifth category of composite measures. Evidence of reliability and validity of each instrument is tracked and reported, and authors report a substantial degree of variability in the reporting of evidence related to validity. Results do not report on patterns in the use of instruments according to intervention types or environmental contexts.

In 2 recent reviews, authors documented nature-based interventions and associated health and well-being outcomes. Wilkie and Davidson [18] examined 52 studies including a categorization of environmental settings, exposure times, and theoretical frameworks. They also report on targeted behaviors and outcomes, which includes mental health and well-being in 79% of studies reviewed and physiological health outcomes in 63% of studies. Results track specific outcomes (eg, self-esteem) that were measured but no details around measurement instruments. Nejade et al [2] similarly reviewed 39 papers that provided evidence of mental and physical health outcomes from nature-based health interventions. The forms of natural outdoor environments included green spaces, blue spaces, and mixed green-blue spaces, ranging from urban parks to wetlands, national parks, or reserves (n=2). The study provides a categorization of nature-based health interventions and activities, reports the mental and physical effects of engagement with natural outdoor environments, and discusses barriers and enablers of such engagement. Instruments used to assess health and well-being outcomes are not assessed.

Despite the proliferation of studies revealing the health and well-being benefits associated with nature contact, there is a clear need to identify and understand the specific instruments being used to assess these benefits. This is especially true in the context of nature contact in parks and other forms of protected areas, where rapid growth in visits to such areas is occurring alongside unprecedented national commitments to protect land or freshwater and marine area the world over. Research is needed to identify the most effective tools for assessing health and well-being outcomes vis-a-vis diverse research contexts (eg, types of environments, activities, and sociodemographic considerations) so that evidence-based policies and guidelines, as well as program outcomes, can be assessed consistently and effectively.

## Methods

### Research Design and Guiding Frameworks

This scoping review has been registered with the Open Science Framework [21] and developed in accordance with the PRISMA-ScR (Preferred Reporting Items for Systematic Reviews and Meta-Analyses extension for Scoping Reviews) guidelines [22]. The completed PRISMA-P checklist is available in Multimedia Appendix 1.

Scoping reviews are broader in nature than systematic reviews, allowing researchers to examine the extent, range, and nature of research activity in a chosen area as opposed to finding the best evidence possible to a tightly defined research question [23]. A scoping review was considered the most appropriate method to address the research objectives due to the capacity to answer broad questions and summarize findings to identify gaps in the literature [2,24]. The scoping review applies Arksey and O’Malley’s [25] five-stage process by (1) identifying the research questions, (2) identifying relevant studies, (3) selecting for studies in the final review, (4) charting the data, and (5) collating, summarizing, and reporting the results. Levac et al [26] expands on this framework to include a sixth optional consulting stage, which the research team deemed unnecessary for the purpose of this study.

Each stage of the scoping review was guided by the Population, Concept, and Context (PCC) framework to establish cohesion between the research questions, search strategy, and inclusion criteria (Textbox 1). The PCC framework is recommended for scoping reviews as a less restrictive alternative to the Population, Intervention, Comparator, and Outcome framework typically applied to systematic reviews [27]. Throughout this process, the purpose of the scoping review will be referred to, and critically discussed by the research team, to ensure that all decisions align with the research objectives and, ultimately, inform both research and practice.

**Developing a scoping review protocol with Population, Concept, and Context.**

- P (Population): adults (aged 18 years or older)
- C (Concept): mental health, subjective well-being, emotional health, psychological health, restoration, coping, attention, mood, and indigenous well-being (including spirituality)
- C (Context): direct contact with parks and protected areas (spatial scope)

### Search Strategy

A comprehensive search strategy was developed by a multidisciplinary team of 6 researchers in collaboration with an experienced university librarian. Following the recommendations of the Joanna Briggs Institute [28] for developing a search strategy, a limited preliminary search was conducted in Google Scholar using keywords related to the research objectives that included “mental health,” “mental well-being,” and “protected areas.” This preliminary search produced 22 peer-reviewed papers that were deemed relevant to the review topic. The titles and abstracts of each paper were screened, along with the key terms used to describe the papers. This preliminary review allowed the research team to identify a robust set of key terms for the search strategy. The full literature index used to develop the search terms for this scoping review can be found in Multimedia Appendix 2. The protected areas terminology used in the literature search reflects the internationally recognized IUCN definition of protected areas and protected area categories, including specialized applications (ie, marine-protected areas). In addition, to inform the development of the search strategy, a search in the APA (American Psychological Association) Dictionary of Psychology identified relevant terms related to mental health, well-being, and subjective well-being.

Searches were conducted in 8 scholarly databases (PubMed, Web of Science, PsycINFO [via ProQuest], ERIC [via EBSCOhost], CINAHL [via EBSCOhost], GreenFILE [via EBSCOhost], OVID, and GEOBASE) known to contain journals focusing on human health and the natural environment. The search hedge contained terms related to domains of protected areas, nature engagement or exposure, and human mental health and well-being (Table 2). The search targets the intersection of environmental settings (protected area), with actions or events (nature engagement or exposure), and associated outcomes (mental health and well-being). Protected areas are recognized not only as spaces designated for conservation but also as venues for nature engagement. There is substantial evidence linking nature engagement with improved mental health and well-being, including benefits associated with protected areas [8,29-32]. The search terms were grouped by the Boolean operator “OR” to enhance the accuracy and relevance of results by accounting for as many concepts as possible and then combined using the Boolean operator “AND” to ensure that only relevant literature that contains all listed search concepts would be generated. The proximity operator “NEAR/20” was used to identify terms within 20 words of each other, regardless of their order. To promote transparency and replicability in future research, the full search strategy for each of the 8 databases is available in Multimedia Appendix 3.

All database searches were conducted on January 4, 2023. To limit the scope of the searches, the selected databases were filtered to include only peer-reviewed journal papers published in English. No date filters were used to limit results to ensure that all relevant studies were included. By excluding a date limiter, the search strategy is more likely to identify trends over time, for instance, when certain instruments were first used to measure mental health and well-being in a parks and protected areas context.

**Table 2:** Primary search hedge subsequently adapted by scholarly database.

Concept	Search terms
Protected area classification	“protected area*” OR “national park*” OR “conserv* area*” OR “provincial park*” OR “state park*” OR “wildlife area*” OR “wildlife sanctuar*” OR “tribal park*” OR “nature reserve*” OR “marine reserve*” OR “marine sanctuar*” OR “conserv* territor*” OR “protected landscape*” OR “protected seascape*” OR “habitat management area*” OR “species management area*” OR “natural area*” OR “wilderness” NEAR/20
Nature engagement	exposure OR access* OR time OR engag* OR visit* OR being OR activity OR exercis* OR experience* AND
Mental health and well-being	“well-being*” OR “psychological restoration” OR “psychological health” OR “restorative*” OR “life satisfaction” OR coping OR “stress hormone” OR cortisol OR “mental health” OR “subjective well*” OR cognit* OR stress* OR emotion* OR anxiety* OR anxious* OR depress* OR mood* OR “state of mind” OR “frame of mind” OR brain* OR mind* OR “self-esteem”

### Study Selection Process

All studies identified by our search strategy were uploaded into the reference manager software Zotero (Corporation for Digital Scholarship) [33]. Study details were then imported into the scoping review software Covidence (Veritas Health Innovation Ltd) [34] where duplicates were removed. The screening (and data extraction process) was piloted by 2 independent reviewers (JB and RB). These reviewers screened a random sample of 20 sources to ensure relative consistency and understanding of the proposed inclusion or exclusion criteria.

All studies were screened by 2 independent reviewers (CER and RB) at 2 levels. At the first level, the title, keywords, and abstracts of each source were assessed against the following criteria listed in Textbox 2. Where both reviewers agreed based on explicit content that a criterion was not met, the study was removed. Where there was disagreement or a lack of explicit content to make a judgement, the study moved to level 2 for full text review using the same inclusion or exclusion criteria.

**Inclusion and exclusion criteria.**

**Inclusion criteria**

- Relevant to the research questions.
- Measures mental health and well-being.
- Protected area context.
- Focuses on direct contact (being physically present) with protected areas.
- Peer-reviewed papers (accessible for retrieval).
- Focuses on adults (aged 18 years or older).
- Available in English.

**Exclusion criteria**

- Not relevant to the research questions.
- Measures only other forms of health and well-being (physical, social, etc).
- Nonprotected area context.
- Focuses on nondirect forms of contact with protected areas (virtual reality, photograph viewing, etc).
- Books, book chapters, or reviews: conference proceedings, dissertations, theses, systematic or scoping reviews, gray literature, news articles, social media content, opinion papers, and inaccessible peer-reviewed papers.
- Includes children (younger than 18 years).
- Not available in English.

At the level of full-text screening, the research team attempted to retrieve the full-text files of all potentially relevant studies through available university library services. If unable to retrieve the full text through the library services, a member of the research team contacted the corresponding authors to obtain a full-text file. Sources that remained unavailable were removed from the review. Once again, 2 independent reviewers (JB and RB) screened the full-text files applying the same inclusion or exclusion criteria that were used at level 1. Disagreements between reviewers at each stage of the selection process were addressed through discussion, involving a third reviewer to resolve conflicts as necessary.

### Data Extraction and Analysis

Data will be extracted using Covidence by 2 independent reviewers (JB and RB) and compared for quality assurance to reduce bias. Any discrepancies that arise will be discussed and conflicts will be resolved by a third reviewer (CER). The proposed data extraction form (Table 3) will be used to identify and extract relevant variables that best address the research objectives. Extracted data from each paper will include descriptive information (eg, author names, title, and year of publication) and study methodology (eg, location, study design, sample, measurement instrument, and timing). Data pertaining to any quantitative or qualitative instruments used to measure mental health and well-being outcomes will also be extracted (eg, dimensions of well-being measured, instrument name, number of items, response scale, and end user engagement). Where a study includes more than 1 instrument of interest, each instrument will be recorded separately.

**Table 3:** Proposed data extraction template, indicating fields for which researchers will extract data with sample outputs.

Domains	Data extraction fields	Sample outputs
Characteristics of the study	Reference Study location Protected area designation Study design Measurement form Timing of measurement Time spent in nature	Full citation of listed study Canada National Park Mixed methods, etc Questionnaire, interview, etc While in protected area Two days, 1 week, etc
Details provided about the quantitative instruments in each study	Dimensions of well-being Instrument name Source Number of items Scale size Substantive validity Structural validity External validity	Affect (eg, feeling, emotion, attachment, or mood) Positive and Negative Affect Schedule (PANAS) Author (year) 20 items 5-point scale n=1 (100%) n=1 (100%) n=0 (0%)
Details provided about the qualitative instruments in each study	Dimensions of well-being Instrument name Source “End user” engagement Multiple researchers involved in theming process Member checks	Affect (eg, feeling, emotion, attachment, or mood) Semistructured interview Author (year) n=1 (100%) n=0 (0%) n=0 (0%)

Drawing on validity criteria from Simms [35], information that the researchers provide about the quantitative measurement instruments in the “Methods” section of each study will be assessed for 3 aspects of construct validity: substantive validity, structural validity, and external validity. Substantive validity is information about whether a measure is theoretically linked to the construct being studied. Structural validity is information about the degree to which the scores of a scale are an adequate indication of what the items measure, while external validity is information about whether the study findings can be generalized to other contexts [35,36]. Each study will be scored as either yes (1), the validity information was provided, or no (0), the validity information was not provided.

A quality appraisal will also be conducted on qualitative measurement instruments used in reviewed studies. This appraisal indicates whether multiple reviewers were involved in the theming process and checks were performed. Similar to the quantitative instruments, each criterion will be scored as yes (1) or no (0) based on whether studies provide evidence that these activities were incorporated into the methodology.

## Results

**Figure 1:**
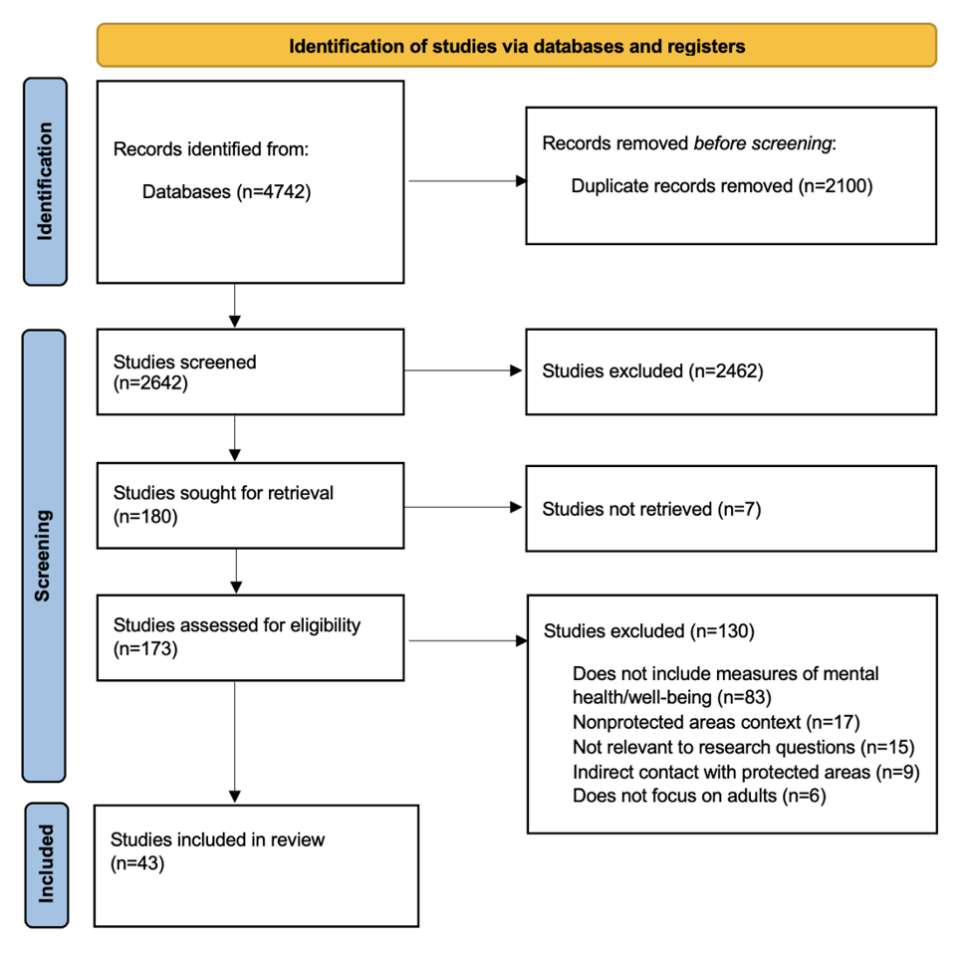
PRISMA flow diagram of the study selection process showing results and exclusions.

Preliminary results of the study selection process are reported here using a PRISMA diagram (Figure 1) in accordance with PRISMA-ScR guidelines [28]. Searches in the 8 scholarly databases on January 4, 2023, yielded an initial 4742 studies (1 merged). From these initial studies, 2642 unique studies were identified for title and abstract screening, after the removal of 2100 duplicates. Through title and abstract screening, 180 studies were selected for full-text review, eliminating 2462 studies as they did not meet the previously outlined inclusion criteria. The full-text papers were sought for retrieval (7 were unavailable), resulting in 173 studies assessed for eligibility through the full-text review process. A total of 43 papers were selected for the final analysis. This pool is larger than those in other scoping reviews on adjacent topics [1], which affects the scope and time frame of the analysis. An updated search was conducted on December 11, 2024, yielding an additional 8 papers to be included in the analysis, for a total of 51 papers. The results of the final scoping review and submission of a manuscript for peer review are expected in April 2025.

## Discussion

### Overview

This protocol details the methodology for a scoping review of the instruments that measure health and well-being linked to nature contact in protected areas, including their psychometric properties. Existing reviews have focused primarily on constructs related to mental health and well-being of adult populations associated with nature contact, although none specifically identify parks or protected areas. The volume of studies retrieved through the selection process suggests a robust evidence base regarding the health and well-being benefits of nature contact, with a likely diversity of measurement tools used. The review will identify, evaluate, and compare measures to provide a comprehensive overview of the quantitative and qualitative methodological instruments and tools used in research to date.

### Strengths and Limitations

The scoping review is subject to limitations, in that it does not include gray and white literature, as well as studies that are not available in English. Given this, some relevant sources may be missed. Peer-reviewed papers not indexed in the searched databases may also be missed, but this limitation was deemed acceptable, given the need to manage the scope of the project. In addition, the extent to which the psychometric properties of an instrument can be evaluated is limited to the information provided within the included studies, which may be lacking descriptions of scale development. A deeper investigation would require looking elsewhere for additional resources, which is beyond the scope of this review. Nevertheless, this review will provide a strong evidence base on which to build future research.

### Conclusions

To the authors’ knowledge, this will be the first scoping review undertaken on measures used to assess mental health and well-being outcomes related to nature contact in a parks and protected areas context. Findings will aid in identifying the strengths and weaknesses of current measurement approaches to mental health and well-being outcomes of nature contact and may be used to guide future research on this topic. For example, nature prescriptions—a health care program comprising written directives by health professionals for visits to natural settings (either individually or in groups) relying heavily on parks and other forms of protected areas—now exist in at least 6 countries [37]. Canada has more than 12,000 health care professionals prescribing nature in parks, and China’s national health strategy includes a commitment to build more than 1000 forest therapy facilities nationwide [38].

Given the unprecedented interest and growth in nature-based health care commitments, it will be necessary to identify and use methods that effectively consider contextual factors. Relevant factors can include demographics (age, gender, and ethnicity) and activities, durations, and environments prescribed. All of these factors must be documented to best ensure reliability and validity when evaluating the outcomes (or benefits) and efficacy of nature prescription programs. This proposed review is also very timely, given the projected growth in the global estate of protected areas as per the United Nations Convention on Biological Diversity Kunming-Montreal Global Biodiversity Framework (detailed in the “Introduction” section).

Adherence to the PRISMA-ScR guidelines will ensure that the findings of this scoping review are of high quality and replicable. Furthermore, by providing insights into the validity of the measurement instruments used, we provide an opportunity to strengthen the methodological quality of future studies. The outlined scoping review will have significant implications for researchers, policy makers, and practitioners working at the nature conservation and human health interface. This review will provide a means to both understand previous research and undertake innovative research initiatives related to mental health and well-being outcomes associated with nature contact in parks and protected areas.

## Appendix

Multimedia Appendix 1 PRISMA-P (Preferred Reporting Items for Systematic review and Meta-Analysis Protocols) 2015 checklist: recommended items to address in a systematic review protocol.

Multimedia Appendix 2 Index of relevant articles that informed the development of the search strategy.

Multimedia Appendix 3 Search strategy for 8 scholarly databases.

## Abbreviations

APA: American Psychological Association
IUCN: International Union for Conservation of Nature
PCC: Population, Concept, and Context
PRISMA-ScR: Preferred Reporting Items for Systematic Reviews and Meta-Analyses extension for Scoping Reviews

## Data Availability

The datasets generated and/or analyzed during this study are available from the corresponding author on reasonable request. The datasets will be reported in the final scoping review manuscript.
