# Supplementary material for: Measuring Adult Health and Well-Being Outcomes Associated With Nature Contact in Parks and Other Forms of Protected Areas: Protocol for a Scoping Review
Source: JMIR Res Protoc. 2025 Mar 24;14:e63338. doi: 10.2196/63338 (PMC11976172; doi:10.2196/63338)
Supplement: Multimedia Appendix 2 [file resprot_v14i1e63338_app2.doc]

Multimedia Appendix 2

Index of relevant articles that informed the development of the search strategy.

| **Index number** | **Citation** |
| --- | --- |
| 1 | Bowen DJ, Neill JT, Crisp SJR. Wilderness adventure therapy effects on the mental health of youth participants. Eval Program Plann. 2016;58:49-59. doi:10.1016/j.evalprogplan.2016.05.005 |
| 2 | Buckley R. Nature tourism and mental health: parks, happiness, and causation. Journal of Sustainable Tourism. 2020;28(9):1409-1424. doi:10.1080/09669582.2020.1742725 |
| 3 | Cini F, Kruger S, Ellis S. A model of intrinsic and extrinsic motivations on subjective well-being: The experience of overnight visitors to a national park. Appl Res Qual Life. 2013;8(1):45-61. doi:10.1007/S11482-012-9173-Y/FIGURES/3 |
| 4 | Farías-Torbidoni EI, Mas-Alòs S, Gil-Moreno-de-Mora G, et al. Health and well-being in protected natural areas— visitors’ satisfaction in three different protected natural area categories in Catalonia, Spain. Int J Environ Res Public Health. 2020;17(18):1-21. doi:10.3390/ijerph17186746 |
| 5 | Ferraro DM, Miller ZD, et al. The phantom chorus: birdsong boosts human well-being in protected areas. Proceedings of the Royal Society B: Biological Sciences. 2020;287:1-9. doi:10.1098/rspb.2020.1811 |
| 6 | Gabrielsen LE, Eskedal LT, Mesel T, et al. The effectiveness of wilderness therapy as mental health treatment for adolescents in Norway: a mixed methods evaluation. Int J Adolesc Youth. 2019;24(3):282-296. doi:10.1080/02673843.2018.1528166 |
| 7 | Garcia Rodrigues J, Villasante S, Pinto IS. Non-material nature’s contributions to people from a marine protected area support multiple dimensions of human well-being. Sustain Sci. 2022;17:793-808. doi:10.1007/s11625-021-01021-x |
| 8 | Harker AL, Stojanovic TA, Majalia AM, Jackson C, Baya S, Dadley Tsiganyiu K. Relationships between livelihoods, well-being, and marine protected areas: Evidence from a community survey, Watamu Marine National Park and Reserve, Kenya. Coastal Management. 2022;50(6):490-513. doi:10.1080/08920753.2022.2126266 |
| 9 | Hausmann A, Toivonen T, Fink C, et al. Understanding sentiment of national park visitors from social media data. People and Nature. 2020;2(3):750-760. doi:10.1002/PAN3.10130/SUPPINFO |
| 10 | Jiricka-Pürrer A, Tadini V, Salak B, Taczanowska K, Tucki A, Senes G. Do protected areas contribute to health and well-being? A cross-cultural comparison. Int J Environ Res Public Health. 2019;16(1172):1-18. doi:10.3390/ijerph16071172 |
| 11 | Kim G, Miller PA. The impact of green infrastructure on human health and well-being: The example of the Huckleberry Trail and the Heritage Community Park and Natural Area in Blacksburg, Virginia. Sustain Cities Soc. 2019;48:1-9. doi:10.1016/J.SCS.2019.101562 |
| 12 | Koss RS, Yotti’ Kingsley J’. Volunteer health and emotional wellbeing in marine protected areas. Ocean Coast Manag. 2010;53:447-453. doi:10.1016/j.ocecoaman.2010.06.002 |
| 13 | Lemieux CJ, Eagles PFJ, Slocombe DS, Doherty ST, Elliott SJ, Mock SE. Human health and well-being motivations and benefits associated with protected area experiences: An opportunity for transforming policy and management in Canada. Parks; 2012; (18)1. doi:10.2305/IUCN.CH |
| 14 | Lemieux CJ, Doherty ST, Eagles PFJ, Groulx MW, Hvenegaard GT, Romagosa F. Policy and management recommendations informed by the health benefits of visitor experiences in Alberta’s protected areas. Journal of Parks and Recreation Administration; 2016; (34)1:24-52. |
| 15 | Li X, Chen C, Wang W, et al. The contribution of national parks to human health and well-being: Visitors’ perceived benefits of Wuyishan National Park-NC-ND. International Journal of Geoheritage and Parks. 2021;9:1-12. doi:10.1016/j.ijgeop.2020.12.004 |
| 16 | Puhakka R, Pitk€ K, Siikam€ Aki P. The health and well-being impacts of protected areas in Finland. Journal of Sustainable Tourism. 2017;25(12):1830-1847. doi:10.1080/09669582.2016.1243696 |
| 17 | Reining CE, Lemieux CJ, Doherty ST. Linking restorative human health outcomes to protected area ecosystem diversity and integrity. Journal of Environmental Planning and Management; 2021; (64)13. doi:10.1080/09640568.2020.1857227 |
| 18 | Romagosa F, Eagles PFJ, Lemieux CJ. From the inside out to the outside in: Exploring the role of parks and protected areas as providers of human health and well-being. Journal of Outdoor Recreation and Tourism; 2015; (10):70-77. doi:10.1016/j.jort.2015.06.009 |
| 19 | Romanillos T, Maneja R, Varga D, Badiella L, Boada M. Protected natural areas: In sickness and in health. Int J Environ Res Public Health. 2018;15(2182). doi:10.3390/ijerph15102182 |
| 20 | Shen J, Hogarth NJ, Hou Y, Duan W. Impact of nature reserves on human well-being – evidence from giant panda reserves in China. J For Econ. 2021;36(1-2):79-101. doi:10.1561/112.00000526 |
| 21 | Wolf ID, Stricker HK, Hagenloh G. Outcome-focused national park experience management: transforming participants, promoting social well-being, and fostering place attachment. Journal of Sustainable Tourism. 2015;23(3):358-381. doi:10.1080/09669582.2014.959968 |
| 22 | Zhou W, Cai J, Chen K. Connecting recreational service to visitor’s well-being: A case study in Qianjiangyuan National Park. Int J Environ Res Public Health. 2022;19(18):11366. doi:10.3390/IJERPH191811366 |
