# Supplementary material for: Measuring Adult Health and Well-Being Outcomes Associated With Nature Contact in Parks and Other Forms of Protected Areas: Protocol for a Scoping Review
Source: JMIR Res Protoc. 2025 Mar 24;14:e63338. doi: 10.2196/63338 (PMC11976172; doi:10.2196/63338)
Supplement: Multimedia Appendix 3 [file resprot_v14i1e63338_app3.doc]

Multimedia Appendix 3

Search strategy for eight scholarly databases

**1. Web of Science (all databases)**

((TS=(("natural area*" OR "wilderness" OR "protected area*" OR "national park*" OR "conserv* area*" OR "provincial park*" OR "state park*" OR "wildlife area*" OR "wildlife sanctuar*" OR "tribal park*” OR “nature reserve*" OR “marine reserve*” OR “marine sanctuar*” OR “conserv* territor*” OR "protected landscape*" OR "protected seascape*" OR "habitat management area*" OR "species management area*") NEAR/20 (Exposure OR Access* OR Time OR Engag* OR Visit* OR Being OR Activity OR Exercis* OR Experience*)))

AND TS=(“well-being*” OR “psychological restoration” OR “psychological health” OR “restorative*” OR “life satisfaction” OR coping OR “stress hormone” OR cortisol OR “mental health” OR “subjective well*” OR cognit* OR stress* OR emotion* OR anxiety* OR anxious* OR depress* OR mood* OR “state of mind” OR “frame of mind” OR brain* OR mind* OR “self-esteem”))

Filters: Exact search: On, English only, and articles only.

**2. CINAHL (through EBSCOHost)**

ABSTRACT SEARCH: ("natural area*" OR "wilderness" OR "protected area*" OR "national park*" OR "conserv* area*" OR "provincial park*" OR "state park*" OR "wildlife area*" OR "wildlife sanctuar*" OR "tribal park*” OR “nature reserve*" OR “marine reserve*” OR “marine sanctuar*” OR “conserv* territor*” OR "protected landscape*" OR "protected seascape*" OR "habitat management area*" OR "species management area*") N20 (Exposure OR Access* OR Time OR Engag* OR Visit* OR Being OR Activity OR Exercis* OR Experience*)

AND ABSTRACT SEARCH: “well-being*” OR “psychological restoration” OR “psychological health” OR “restorative*” OR “life satisfaction” OR coping OR “stress hormone” OR cortisol OR “mental health” OR “subjective well*” OR cognit* OR stress* OR emotion* OR anxiety* OR anxious* OR depress* OR mood* OR “state of mind” OR “frame of mind” OR brain* OR mind* OR “self-esteem”

Filters: Academic journals, peer-reviewed, English only.

**3. ERIC (through EBSCOHost)**

ABSTRACT SEARCH: ("natural area*" OR "wilderness" OR "protected area*" OR "national park*" OR "conserv* area*" OR "provincial park*" OR "state park*" OR "wildlife area*" OR "wildlife sanctuar*" OR "tribal park*” OR “nature reserve*" OR “marine reserve*” OR “marine sanctuar*” OR “conserv* territor*” OR "protected landscape*" OR "protected seascape*" OR "habitat management area*" OR "species management area*") N20 (Exposure OR Access* OR Time OR Engag* OR Visit* OR Being OR Activity OR Exercis* OR Experience*)

AND ABSTRACT SEARCH: “well-being*” OR “psychological restoration” OR “psychological health” OR “restorative*” OR “life satisfaction” OR coping OR “stress hormone” OR cortisol OR “mental health” OR “subjective well*” OR cognit* OR stress* OR emotion* OR anxiety* OR anxious* OR depress* OR mood* OR “state of mind” OR “frame of mind” OR brain* OR mind* OR “self-esteem”

Filters: Academic journals, peer-reviewed, English only.

**4. GreenFILE (through EBSCOHost)**

ABSTRACT SEARCH: ("natural area*" OR "wilderness" OR "protected area*" OR "national park*" OR "conserv* area*" OR "provincial park*" OR "state park*" OR "wildlife area*" OR "wildlife sanctuar*" OR "tribal park*” OR “nature reserve*" OR “marine reserve*” OR “marine sanctuar*” OR “conserv* territor*” OR "protected landscape*" OR "protected seascape*" OR "habitat management area*" OR "species management area*") N20 (Exposure OR Access* OR Time OR Engag* OR Visit* OR Being OR Activity OR Exercis* OR Experience*)

AND ABSTRACT SEARCH: “well-being*” OR “psychological restoration” OR “psychological health” OR “restorative*” OR “life satisfaction” OR coping OR “stress hormone” OR cortisol OR “mental health” OR “subjective well*” OR cognit* OR stress* OR emotion* OR anxiety* OR anxious* OR depress* OR mood* OR “state of mind” OR “frame of mind” OR brain* OR mind* OR “self-esteem”

Filters: Academic journals, peer-reviewed, English only.

**5. PsychInfo (through ProQuest)**

All abstract & summary text - SUMMARY: ("natural area*" OR "wilderness" OR "protected area*" OR "national park*" OR "conserv* area*" OR "provincial park*" OR "state park*" OR "wildlife area*" OR "wildlife sanctuar*" OR "wilderness area*" OR "tribal park*" OR "nature reserve*" OR "marine reserve*" OR "marine sanctuar*" OR "conserv* territor*" OR "protected landscape*" OR "protected seascape*" OR "habitat management area*" OR "species management area*") NEAR/20 (Exposure OR Access* OR Time OR Engag* OR Visit* OR Being OR Activity OR Exercis* OR Experience*)

AND All abstract & summary text - SUMMARY: "well-being*" OR "psychological restoration" OR "psychological health" OR "restorative*" OR "life satisfaction" OR coping OR "stress hormone" OR cortisol OR "mental health" OR "subjective well*" OR cognit* OR stress* OR emotion* OR anxiety* OR anxious* OR depress* OR mood* OR "state of mind" OR "frame of mind" OR brain* OR mind* OR "self-esteem"

Filters: Peer-reviewed journals, English only

**6. Ovid (all resources)**

((("natural area*" or "wilderness" or "protected area*" or "national park*" or "conserv* area*" or "provincial park*" or "state park*" or "wildlife area*" or "wildlife sanctuar*" or "tribal park*" or "nature reserve*" or "marine reserve*" or "marine sanctuar*" or "conserv* territor*" or "protected landscape*" or "protected seascape*" or "habitat management area*" or "species management area*") adj20 (Exposure or Access* or Time or Engag* or Visit* or Being or Activity or Exercis* or Experience*))

AND ("well-being*" or "psychological restoration" or "psychological health" or "restorative*" or "life satisfaction" or coping or "stress hormone" or cortisol or "mental health" or "subjective well*" or cognit* or stress* or emotion* or anxiety* or anxious* or depress* or mood* or "state of mind" or "frame of mind" or brain* or mind* or "self-esteem")).ab.

Filters: Peer-reviewed journals, English language

**7. PubMed**

(("natural area*"[Title/Abstract] OR "wilderness"[Title/Abstract] OR "protected area*"[Title/Abstract] OR "national park*"[Title/Abstract] OR "conserv* area*"[Title/Abstract] OR "provincial park*"[Title/Abstract] OR "state park*"[Title/Abstract] OR "wildlife area*"[Title/Abstract] OR "wildlife sanctuar*"[Title/Abstract] OR "tribal park*"[Title/Abstract] OR "nature reserve*"[Title/Abstract] OR "marine reserve*"[Title/Abstract] OR "marine sanctuar*"[Title/Abstract] OR "conserv* territor*"[Title/Abstract] OR "protected landscape*"[Title/Abstract] OR "protected seascape*"[Title/Abstract] OR "habitat management area*"[Title/Abstract] OR "species management area*"[Title/Abstract])

AND (Exposure[Title/Abstract] OR Access*[Title/Abstract] OR Time[Title/Abstract] OR Engag*[Title/Abstract] OR Visit*[Title/Abstract] OR Being[Title/Abstract] OR Activity[Title/Abstract] OR Exercis*[Title/Abstract] OR Experience*[Title/Abstract]))

AND ("Mental Health" [Mesh] OR “Psychological Well-Being" [Mesh] OR “Stress Disorders, Traumatic, Acute” [Mesh] OR “Anxiety” [Mesh] OR “Emotions” [Mesh] OR “Brain” [Mesh] OR “Self Concept” [Mesh] OR "well-being*" OR "psychological restoration" OR "psychological health" OR "restorative*" OR "life satisfaction" OR coping OR "stress hormone" OR cortisol OR "mental health" OR "subjective well*" OR cognit* OR stress* OR emotion* OR anxiety* OR anxious* OR depress* OR mood* OR "state of mind" OR "frame of mind" OR brain* OR mind* OR "self-esteem")

Filters: English only

**8. GEOBASE**

(((((("natural area*" OR "wilderness" OR "protected area*" OR "national park*" OR "conserv* area*" OR "provincial park*" OR "state park*" OR "wildlife area*" OR "wildlife sanctuar*" OR "tribal park*" OR "nature reserve*" OR "marine reserve*" OR "marine sanctuar*" OR "conserv* territor*" OR "protected landscape*" OR "protected seascape*" OR "habitat management area*" OR "species management area*") WN KY)

AND ((Exposure OR Access* OR Time OR Engag* OR Visit* OR Being OR Activity OR Exercis* OR Experience*) WN KY))

AND (("well-being*" OR "psychological restoration" OR "psychological health" OR "restorative*" OR "life satisfaction" OR coping OR "stress hormone" OR cortisol OR "mental health" OR "subjective well*" OR cognit* OR stress* OR emotion* OR anxiety* OR anxious* OR depress* OR mood* OR "state of mind" OR "frame of mind" OR brain* OR mind* OR "self-esteem") WN KY)))

AND (({ja} WN DT)

AND ({english} WN LA)))

Filters: Journal articles only, English only.
